# Revisiting the Relationship Between Alzheimer’s Disease and Cancer With a circRNA Perspective

**DOI:** 10.3389/fcell.2021.647197

**Published:** 2021-03-11

**Authors:** Danze Chen, Shijia Hao, Jianzhen Xu

**Affiliations:** ^1^Computational Systems Biology Lab, Shantou University Medical College (SUMC), Shantou, China; ^2^Guangdong Provincial Key Laboratory for Diagnosis and Treatment of Breast Cancer, Shantou University Medical College (SUMC), Shantou, China

**Keywords:** circRNA, cancer, Alzheimer’s disease, inflammation, systems biology

## Abstract

**Background:**

Increasing evidence indicates an association between the incidence of Alzheimer’s disease (AD) and cancer development. Despite advances being made by comparisons from epidemiological studies, common pathways and molecular mechanisms, little is known about the identities of the circular RNAs (circRNAs) involved in the development and progression of these two pathologies and their possible correlations. The aim of this study was to explore the circRNA relationship between AD and cancer.

**Materials and Methods:**

In this investigation, circRNAs that were significantly dysregulated in AD or associated with AD diagnosis, clinical dementia severity, and neuropathological severity, were examined in a large panel of 28 cancer types. On the basis of shared abnormal circRNAs in AD and cancers, we constructed a circRNA-micro RNA (miRNA)-messenger RNA (mRNA) network by leveraging experimentally identified miRNA-circRNA and miRNA-mRNA interactions from crosslinking-immunoprecipitation sequencing data.

**Results:**

An inverse correlation of expression pattern was found in acute myeloid leukemia, juvenile myelomonocytic leukemia, renal cell carcinoma, and myelofibrosis. CircRNAs associated with AD diagnosis and clinical severity demonstrated negative correlation in more cancer types. Notably, differentially expressed candidate circRNAs in temporal lobe epilepsy were not associated with any cancers. Gene Ontology and KEGG pathway analysis suggested the circRNA-regulated genes are significantly associated with interleukin-12-mediated signaling and viral response. CircPICALM, circRTN4 and circMAN2A1 are the hub nodes in the circRNA-miRNA-target network.

**Conclusion:**

Our results indicated the relevance of inflammation signaling as a common pathogenesis shared by cancer and AD and provided novel insight for therapeutics targeting circRNAs.

## Introduction

Alzheimer’s disease (AD) is a chronic progressive neurodegenerative disease that affects millions of people worldwide (Lane et al., 2018). Accumulating evidence suggests a biological link between cancer and AD neuropathology (Snyder et al., 2017). Both are age-associated diseases; although one is degenerative and other is over-proliferative, the risks of both increase significantly with age (Childs et al., 2015; Xia et al., 2018). Furthermore, both diseases are complex and heterogeneous, which greatly challenges accurate diagnosis and efficient treatment (Dagogo-Jack and Shaw, 2018; Nikolac Perkovic and Pivac, 2019). Several epidemiological investigations and meta-analyses suggested a possible inverse relationship between the incidences of these pathologies (Musicco et al., 2013; Zhang et al., 2015). Consistent with this observation, AD is associated with increased cellular death and decreased proliferative signaling, whereas uncontrolled proliferation and apoptosis inhibition are the hallmarks in cancers (Hanahan and Weinberg, 2011; Nudelman et al., 2019). Therefore, AD and cancer may potentially share some regulatory factors, but with opposite regulation direction. In addition, Feng et al. (2017) detected significant genetic correlations between AD and certain types of cancer from genome-wide association study datasets, which indicates the presence of shared genetic variants and disease mechanisms.

Supporting evidence also emerged from molecular studies. For example, several systemic reviews proposed sets of protein-coding genes and mechanistic links among them that play an important role in both cancer and AD (Shafi, 2016; Snyder et al., 2017). Holohan et al., reviewed the functional microRNAs (miRNAs) in both diseases and identified eight (miR-9,-29a/b,-101,-107,-125b,-146a,-153,and-195) that interconnect the two conditions (Holohan et al., 2012). Recently, Battaglia et al. (2019) integrated text mining resources, protein-protein interactions, and gene-miRNA interactions to reveal the complex regulatory mechanisms of gene expression in AD and cancer.

Circular RNA (circRNA) is a circular form of the non-coding RNA family that has been widely implicated in neurodegenerative disorders, cardiovascular diseases, and carcinogenesis (Aufiero et al., 2019; Dube et al., 2019; Zhao et al., 2019). For example, we recently reported that circTADA2A can act as a miRNA decoy, thus affecting its effector SOCS3 to suppress cell migration, invasion, and clonogenicity in breast cancer (Xu et al., 2019). Another study found that one highly represented circRNA in the human brain (CDR1as) mutually interacted with miRNAs such as miRNA-7 and miRNA-671, to establish a sophisticated regulatory network (Kleaveland et al., 2018). Interestingly, mice without the CDR1as locus displayed neuropsychiatric disorder-like symptoms (Piwecka et al., 2017).

Although circRNA is intimately connected with both AD and cancers, there have been few studies on circRNA expression until now. Recent large-scale circRNA deep sequencing studies in AD and a variety of cancers provide strong data to rigorously assess their relationship. Here, we developed a quantitative approach to inspect the expression patterns of the AD-associated circRNAs in 28 cancer types. Temporal lobe epilepsy (TLE) is another common chronic neurological disorder and was examined for comparison. In order to deeply understand the biological mechanisms, we constructed a circRNA-miRNA-target network to identify common changed pathologies underlying AD and cancer.

## Materials and Methods

### RNA-Seq Data

Cancer circRNA sequencing data was downloaded from the MiOncoCirc project, which characterized circRNAs expression across > 2,000 tumor tissues (Vo et al., 2019). CircRNA reads of control tissue and multiple cancer tissues were used for differential expression analysis. Cortical circRNA expression datasets from the Knight Alzheimer Disease Research Center (Knight ADRC) and the independent Mount Sinai Brain Bank (MSBB) were generated from individuals with and without AD and used in this study (Wang et al., 2018; Dube et al., 2019). TLE-associated circRNA datasets termed TLE1 and TLE2 were from Li et al. (2018) and Mills et al. (2020), respecttively.

### Correlation Analysis

CircRNAs that are differentially expressed in neuropathological AD case–control, or correlated with two clinical traits: Braak score and Clinical Dementia Rating (CDR) were adopted from the above-mentioned AD datasets, respectively. Similarly, the corresponding log2 fold change (Log2FC) values were derived from the two TLE datasets. For the MiOncoCirc cancer dataset, we filtered the cancer samples with a minimum of 5 tumor samples and combined them with 25 normal controls to form 28 comparison groups corresponding to different cancer types (Supplementary Table 1). Log2FC for each circRNA was computed by DESeq2 software with the same parameters used for AD and TLE analysis (Love et al., 2014). Finally, Spearman’s correlation tests were performed to identify associations between AD and each types of different cancer based on log2 FC. Similar correlations were also calculated for TLE-associated circRNAs with cancer data.

### Construction of circRNA-miRNA-Target Network

Seventeen circRNAs that were significantly associated with all the three AD traits such as Braak score, CDR and differential expressed were included in the follow-up analysis. To identify mRNAs potentially regulated by circRNAs through miRNAs, the ENCORI Argonaut (AGO)-crosslinking-immunoprecipitation sequencing data (CLIP-seq) database was queried (Li et al., 2014). ENCORI is an open-source platform for studying the miRNA-ncRNA and miRNA-mRNA interactions from CLIP-seq, degradome-seq, and RNA-RNA interactome data. Using curl commands to access ENCORI API, the miRNA-mRNA and miRNA-mRNA interaction data were collected. For the reliability of the circRNA-miRNA-target network, a CLIP-seq experimental evidence > 1 threshold was used to filter out spurious interactions.

### Gene Ontology (GO) Enrichment Analysis and Network Analysis

The Python package gseapy was applied on mRNAs regulated by 17 circRNAs for identifying GO biological processes and KEGG pathways^1^. GO and KEGG enrichment analysis results were plotted with R package ggplot2^2^. Gene annotated in interleukin (IL)-12 signaling pathway and viral process, together with the associated miRNAs and circRNAs are used to construct the circRNA-miRNA-mRNA interaction network. This network was plotted with Cytoscape (Shannon et al., 2003). To assess the centrality of each node in this network, four global parameters (Closeness, Betweenness, Stress and BottleNeck) and one local parameter (Degree), were computed in Cytoscape plugin cytoHubba. For the definitions and methods to compute these network topological scores, please refer to (Chin et al., 2014).

## Results

### circRNA Expression Is Inversely Correlated Between AD and Some Cancers

Dube et al. (2019) previously conducted a cortical circRNA expression survey in 83 AD patients and 13 healthy controls. Using the RNA-seq dataset (Knight ADRC), they identified a set of differentially expressed circRNAs (Dube et al., 2019). They also used the same methods to analyze published, independent RNA-seq data (MSBB) on AD and non-AD groups (Wang et al., 2018). As presented in Supplementary Table 2, 39 and 75 differentially expressed circRNAs were identified from the Knight ADRC and MSBB datasets. For Knight ADRC data, comparing their expression pattern with that in cancer datasets indicated inverse correlations with nine cancer subtypes (Table 1). These correlations were confirmed in acute myeloid leukemia (AML), juvenile myelomonocytic leukemia (JMML), renal cell carcinoma (KDNY), and myelofibrosis (MPN) datasets from the MSBB (Table 1). Braak score is a neurofibrillary pathological measure of AD severity. Clinical Dementia Rating (CDR) is a clinical dementia severity parameter. These two AD quantitative traits were also used to identify associations between circRNA expression and AD diagnosis (Morris, 1993; Braak et al., 2006). When utilizing 33 candidate circRNAs associated with Braak score from the MSBB data (Supplementary Table 3), significant negative correlations were found in more cancer types (17 of 28), and most were replicate using the Knight ADRC data (Table 1). Similarly, when using CDR to select circRNAs (Supplementary Table 4), inverse associations were still found in 11 cancers (Table 1). Interestingly, stable negative correlations were retained in AML, JMML, KDNY, and MPN, when simultaneously considering circRNAs either differentially expressed or associated with Braak score, and CDR. The circRNA expression pattern was negatively correlated in six cancers, namely, acute lymphoblastic leukemia (ALL), intrahepatic cholangiocarcinoma (CHOL), rectal adenocarcinoma (COLO), esophageal adenocarcinoma (ESCA), head and neck squamous cell carcinoma (HNSC), and non-Hodgkin lymphoma (NHL), if both Braak score and CDR were considered for associated circRNA selection (Table 1). Collectively, these results support a consistent, replicable, and significant association in circRNA expression changes between cancer and AD.

**TABLE 1:** CircRNA expression patterns are negatively correlated in AD and some cancers.

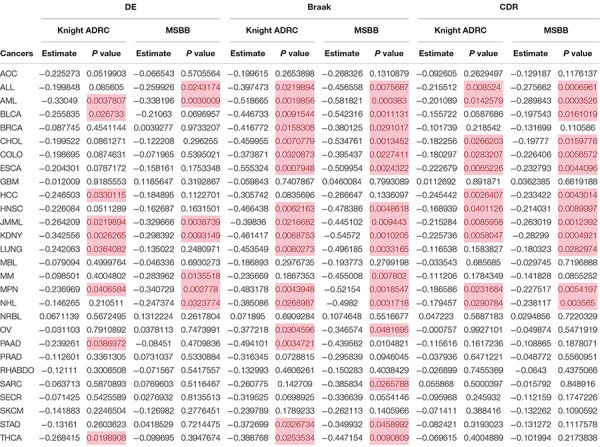

*Shadowed numbers indicate significant correlation with a cutoff *p* < 0.05.*

### Candidate circRNAs in TLE Are Not Correlated With Any Cancers

Since the identified correlations may be due to brain disease in general rather than AD specifically, we leveraged additional two TLE sequencing datasets to rule out this possibility (Li et al., 2018; Mills et al., 2020). In the original TLE RNA-seq projects, there were no quantitative indices to reflect the clinical features, so only differentially expressed circRNAs were identified in the TLE patients. Thirty two and nine DE circRNAs were selected from the TLE1 and TLE 2 datasets, respectively (Supplementary Table 5). As shown in Supplementary Table 6, no associations were found in any cancer types from either TLE dataset when similar correlation analyses were performed.

### Systems Biology Approaches to Identify Common circRNAs and Shared Mechanisms in AD and Cancer

**FIGURE 1:**
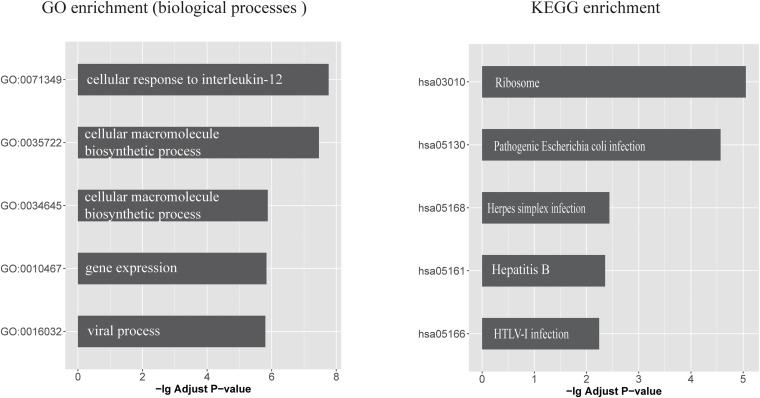
The top five GO biological processes and KEGG pathways that enriched with circRNA-regulated mRNAs **(left)** GO biological processes categories and **(right)** KEGG pathways, which enriched with circRNA-regulated mRNAs. x-axis represents the -log10 (Adjusted P value).

Seventeen circRNAs (circHOMER1, circST18, circMAN2A1, circRTN4, circPICALM, circMAP7, circCORO1C, circDOCK1, circFMN1, circDGKB, circDNAJC6, circERBIN, circRIMS1, circWDR78, circL3MBTL4, circEPB41L5, and circYY1AP1) were significantly associated with all of the three traits (DE, Braak score, and CDR) and were therefore used in the following investigation (False discovery rate < 0.05). Upon examining the CLIP-seq data from the ENCORI database, six circRNAs had direct experimental support for binding with at least one miRNA. These circRNAs target a total of 167 miRNAs that are further linked with 419 mRNAs. The biological consequences of these circRNA-regulated mRNAs were analyzed based on GO annotations and KEGG pathways. Supplementary Table 7 lists all significant GO functional categories and KEGG pathways (adjusted *p* < 0.05). The top five significant functional categories are shown in Figure 1. It can be seen that the most significant GO category is “cellular response to interleukin (IL)-12.“Viral process” is another specific functional category indicated by enrichment analysis. Similarly, circRNA targeting mRNAs are significantly enriched in several KEGG pathways such as “Herpes simplex infection” and “pathogenic Escherichia coli infection.”

**FIGURE 2:**
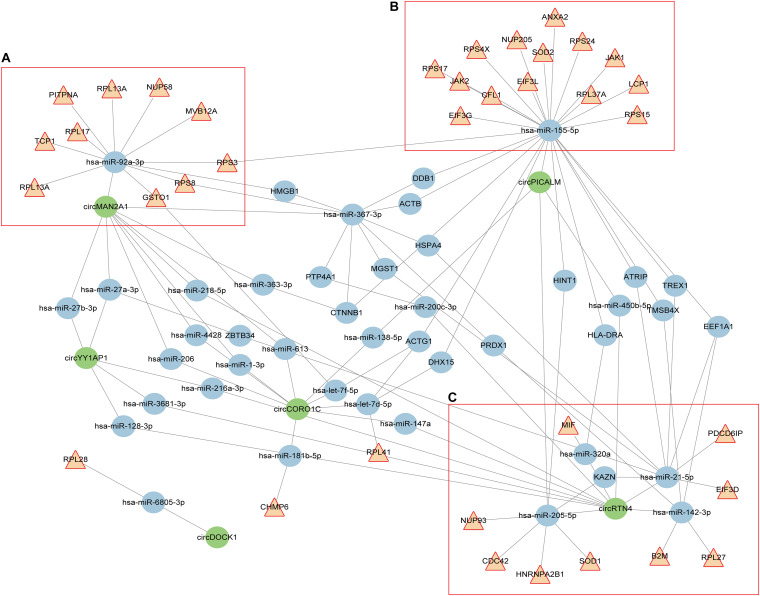
circRNA-miRNA-mRNA interaction network constructed from shared aberrant circRNAs in AD and cancers. Brown triangular nodes represent genes annotated in IL-12 signaling pathways or viral response. circRNAs are colored in green. Edges represent the interactions derived from CLIP data. **(A–C)** Three key clusters that included three highly expressed circRNAs (circPICALM, circMAN2A1, and circRTN4).

For clarity, we extracted the genes annotated in cellular response to interleukin (IL)-12 and viral process categories, and constructed the circRNA-miRNA-mRNA interaction network. As shown in Figure 2, these circRNAs, miRNAs, and mRNAs are highly connected with each other, with only a few outliers. We computed several network parameters to assess the centrality and importance of each node in this network (Supplementary Table 8). circRTN4 and circMAN2A1 are among the top scoring nodes according to all the five parameters. CircPICALM ranks high according to global network properties, but its degree is lower than another circRNA, circCORO1C. This is because both hsa-miR-205-5p and hsa-miR-155-5p are its nearest neighbors. These two miRNAs immediately connected with many inflammation related genes. On the contrary, circCORO1C embedded in a cluster that is away from the inflammation related genes (Figure 2). Generally speaking, the higher score a node has, the more critical position it takes up in the whole network. Thus CircPICALM, circRTN4 and circMAN2A1 were identified as hub nodes in the circRNA-miRNA-target network. In one cluster, hsa-miR-92a-3p links circMAN2A1 with a group of mRNAs (Figure 2A). In the circPICALM cluster, several inflammatory genes surround hsa-miR-155-5p, which is connected with circPICALM (Figure 2B); Finally two circRNAs (circRTN4 and circPICALM), four miRNA (hsa-miR-142-3p, hsa-miR-205-5p, hsa-miR-21-5p and hsa-miR-320a), and nine mRNAs (HNRNPA2B1,SOD1, B2M,RPL27, EIF3D, PDCD6IP, MIF, NUP93, and CDC42) form the last cluster (Figure 2C).

## Discussion

Years of drug discovery for AD have not yielded meaningful success as the precise etiology of AD remains unclear. Elucidating the relationship between cancer and AD in terms of cellular pathways and molecular interactions will help to uncover factors that contribute to the pathogeneses of both diseases. These results may also guide novel therapeutic strategies and suggest potential repurposing of cancer drugs for AD treatment.

Using large-scale circRNA expression datasets from cancer patients, we verified the inverse correlation hypothesis for AD but not TLE. Among the enriched biological processes of genes shared by both diseases, the cellular responses to IL-12 and its mediated signaling pathways are the most significant and specific mechanisms.

The IL-12 cytokine family includes several cytokines such as IL-12, IL-23, IL-27, and IL-35. They play important roles in the development of appropriate immune responses in various disease conditions (Tugues et al., 2015; Yan et al., 2018). In cancer, IL-12 activates multiple antitumor immunity pathways. IL-12 also enhances differentiation of Th1 cells and promotes type II interferon (IFN)- g production. IL-12 and IFN-g can also regulate the tumor microenvironment. Many clinical trials conducted in the last decade with IL-12 alone or in combination with other therapies showed similar or better antitumor efficacy; however, they did not translate into clinical use. The majority of ongoing trials involving IL-12 treatment failed to show sustained antitumor responses, and was associated with toxic side effects. Currently researchers are studying gene therapy approaches to locally deliver IL-12 and avoid side effects (Lasek et al., 2014). On the other hand, the importance of the innate immune response in AD pathogenesis has become clear in recent years. For example, extracellular amyloid-β (Aβ) deposits are a prominent hallmark of AD. Inhibition of IL-12/IL-23 signaling by targeting IL12p40, the common subunit of both IL-12 and IL-23, using either genetic or pharmacological strategies reduced Aβ cerebral load in AD–like APPPS1 mice (Vom Berg et al., 2012). Collectively, our enrichment analysis is consistent with the above reports, suggesting the IL-12/IL-23 axis as a common mechanism in AD and cancer.

Based on both GO terms and KEGG pathway analysis, many circRNAs targeting mRNAs have virus infection related function. It highlights that AD shares a viral etiology with some cancers. While the infectious hypothesis of AD originated decades ago, it has steadily accumulated evidence in recent years. A consensus statement from leading AD experts claimed that herpes virus (HSV1) is the most likely culprit, but HSV2 may also contribute. Indeed, a clinical trial for valacyclovir, the most widely used generic antiviral drug, is actively being investigated in AD patients (Itzhaki et al., 2016).

It should be noted that both the IL-12 pathway and viral process are connected with inflammatory cascades, which are thought to have major impacts in AD. In the so-called inflammation-driven pathogenesis hypothesis of AD, inflammation damage signals such as viral infections activate nuclear factor-Kβ, synthesis and release of proinflammatory cytokines (Guzman-Martinez et al., 2019; Sochocka et al., 2019). Secretion of IL-12 and related cytokines affect neuronal receptors and ultimately lead to Aβ accumulation and tau hyperphosphorylation in neurons. Our results strongly indicated circRNAs participated in these processes. Thus, anti-neuroinflammation pathways, possibly via circRNA or associated miRNAs interfering, may be a good avenue to explore for drug discovery in AD (Guzman-Martinez et al., 2019).

We identified three circRNA (circPICALM, circMAN2A1, and circRTN4), connected with miRNAs and mRNAs to form three clusters in the circRNA-miRNA-mRNA network. All three circRNAs were previously found to be highly expressed in AD patients (Dube et al., 2019), but currently there is only functional study on CircPICALM in cancer. CircPICALM was downregulated in bladder cancer, and its’ low expression was associated with clinicopathological factors such as advanced T stage, high grade, and poor overall survival. Mechanistically, CircPICALM sponges to miR-1265, which further interfering with the miRNA target STEAP4. CircPICALM can inhibit bladder cancer metastasis and regulate epithelial to mesenchymal transition (Yan et al., 2019). In another cluster, circRTN4 connected with four miRNAs (Figure 2C). A report found that hsa-miR-142-3p in serum is lowly expressed thus can be used as biomarker to distinguish AD patients from normal controls (Kumar et al., 2013). Interestingly, two independent groups found elevated hsa-miR-142-3p level in acute leukemias and esophageal squamous cell carcinoma samples (Lin et al., 2012; Dahlhaus et al., 2013). The expression of hsa-miR-142-3p is also associated with clinical data such as overall survival and prognosis. Cholangiocarcinoma is a malignant tumor of bile duct epithelial cells. Similarly, profiling analysis of plasma extracellular vesicles associated miRNAs found hsa-miR-21-5p significantly down-regulated in AD samples respect to dementia with Lewy bodies patients (Gamez-Valero et al., 2019). On the other hand, hsa-miR-21-5p is markedly elevated in hepatocellular carcinoma and gastric cancer (Park et al., 2016; Pu et al., 2018). These results suggested this miRNA can possible use as biomarkers both in AD and cancers. miR-205-5p, another miRNA linked with circRTN4, is the most up-regulated miRNA in cholangiocarcinoma cell-derived exosomes. Inhibition of hsa-miR-205-5p reduced cell invasion and migration (Kitdumrongthum et al., 2018). Unfortunately there is no functional study on the fourth hsa-miR-320a both in cancer and AD. For the circMAN2A1related cluster, we noticed that MAN2A1 (mannosidase alpha class 2A member 1), the parental gene of circMAN2A1, play an important role in glycometabolism. In additional to the classic miRNA mediated functioning, circRNA can also interfere with its parental gene via a variety of mechanisms such as epigenetic control, splicing, transcription, or translation (Zhao et al., 2019). MAN2A1 encodes a glycosyl hydrolase that localizes to the Golgi and catalyzes the final hydrolytic step in the asparagine-linked oligosaccharide (N-glycan) maturation pathway (Moremen and Robbins, 1991). Variants in MAN2A1 have been associated to intelligence and general cognitive ability via GWAS (Savage et al., 2018). Indeed circMAN2A1 is also associated with dementia (Supplementary Table 4), further highlighting the importance of this gene locus in neurodegenerative phenotypes. A recent study found Man2a1-null cancer cells are more sensitive to T cell-mediated tumor killing. This finding has translational relevance since pharmacological inhibition of MAN2A1 by swainsonine synergized with anti-PD-L1 in the treatment of melanoma and lung cancer (Shi et al., 2020). In the future, it would be interesting to explore whether circMAN2A1 participates in MAN2A1regulation, and in which manners circMAN2A1 involves in the regulation.

## Conclusion

Overall, our analysis reveals the negative associations between AD and some type of cancers from the circRNA perspective. Based on systems biology approach, we also identified three circRNAs clusters that potentially interconnect the two conditions. These findings should be validated when more AD omics datasets are available. It also needs to investigate the molecular mechanisms bridging AD and some cancers, with the ultimate goal of developing circRNA-based therapies for both diseases.

## Data Availability Statement

The original contributions presented in the study are included in the article/Supplementary Material, further inquiries can be directed to the corresponding author.

## Author Contributions

JX conceived, designed, and supervised the study, and drafted the manuscript. DC and SH collected the data and performed all data analysis. All authors reviewed and approved the final manuscript.

## Conflict of Interest

The authors declare that the research was conducted in the absence of any commercial or financial relationships that could be construed as a potential conflict of interest.

## Footnotes

1 https://github.com/zqfang/GSEApy

2 https://ggplot2.tidyverse.org/reference/

## Abbreviations

ACC: adrenocortical carcinoma
ALL: acute lymphoblastic leukemia
AML: acute myeloid leukemia
BLCA: bladder urothelial carcinoma
BRCA: breast invasive ductal carcinoma
CHOL: intrahepatic cholangiocarcinoma
COLO: rectal adenocarcinoma
ESCA: esophageal adenocarcinoma
GBM: glioblastoma multiforme
HCC: hepatocellular carcinoma
HNSC: head and neck squamous cell carcinoma
JMML: juvenile myelomonocytic leukemia
KDNY: renal cell carcinoma
LUNG: lung adenocarcinoma
MBL: medulloblastoma
MM: multiple_myeloma
MPN: myelofibrosis
NHL: non-hodgkin lymphoma
NRBL: neuroblastoma
OV: ovarian serous adenocarcinoma
PAAD: pancreatic adenocarcinoma
PRAD: prostate adenocarcinoma
RHABDO: rhabdomyosarcoma
SARC: sarcoma
SECR: adenoid cystic carcinoma
SKCM: skin cutaneous melanoma
STAD: stomach adenocarcinoma
THCA: thyroid carcinoma.

## References

[B1] Aufiero S. Reckman Y. J. Pinto Y. M. Creemers E. E. (2019). Circular Rnas open a new chapter in cardiovascular biology. *Nat. Rev. Cardiol.* 16, 503–514. 10.1038/s41569-019-0185-2 30952956

[B2] Battaglia C. Venturin M. Sojic A. Jesuthasan N. Orro A. Spinelli R. (2019). Candidate genes and MiRNAs linked to the inverse relationship between cancer and alzheimer’s disease: insights from data mining and enrichment analysis. *Front. Genet.* 10:846. 10.3389/fgene.2019.00846 31608105 PMC6771301

[B3] Braak H. Alafuzoff I. Arzberger T. Kretzschmar H. Del Tredici K. (2006). Staging of Alzheimer disease-associated neurofibrillary pathology using paraffin sections and immunocytochemistry. *Acta Neuropathol.* 112 389–404. 10.1007/s00401-006-0127-z 16906426 PMC3906709

[B4] Childs B. G. Durik M. Baker D. J. van Deursen J. M. (2015). Cellular senescence in aging and age-related disease: from mechanisms to therapy. *Nat. Med.* 21 1424–1435. 10.1038/nm.4000 26646499 PMC4748967

[B5] Chin C. H. Chen S. H. Wu H. H. Ho C. W. Ko M. T. Lin C. Y. (2014). cytoHubba: identifying hub objects and sub-networks from complex interactome. *BMC Syst. Biol.* 8(Suppl. 4):S11. 10.1186/1752-0509-8-S4-S11 25521941 PMC4290687

[B6] Dagogo-Jack I. Shaw A. T. (2018). Tumour heterogeneity and resistance to cancer therapies. *Nat. Rev. Clin. Oncol.* 15 81–94. 10.1038/nrclinonc.2017.166 29115304

[B7] Dahlhaus M. Roolf C. Ruck S. Lange S. Freund M. Junghanss C. (2013). Expression and prognostic significance of hsa-miR-142-3p in acute leukemias. *Neoplasma* 60 432–438. 10.4149/neo_2013_056 23581416

[B8] Dube U. Del-Aguila J. L. Li Z. Budde J. P. Jiang S. Hsu S. (2019). An atlas of cortical circular RNA expression in Alzheimer disease brains demonstrates clinical and pathological associations. *Nat. Neurosci.* 22 1903–1912. 10.1038/s41593-019-0501-5 31591557 PMC6858549

[B9] Feng Y. A. Cho K. Lindstrom S. Kraft P. Cormack J. C. T. S. IGAP Consortium, (2017). Investigating the genetic relationship between Alzheimer’s disease and cancer using GWAS summary statistics. *Hum. Genet.* 136 1341–1351. 10.1007/s00439-017-1831-6 28780673 PMC5693762

[B10] Gamez-Valero A. Campdelacreu J. Vilas D. Ispierto L. Rene R. Alvarez R. (2019). Exploratory study on microRNA profiles from plasma-derived extracellular vesicles in Alzheimer’s disease and dementia with Lewy bodies. *Transl. Neurodegener.* 8:31. 10.1186/s40035-019-0169-5 31592314 PMC6775659

[B11] Guzman-Martinez L. Maccioni R. B. Andrade V. Navarrete L. P. Pastorm M. G. Ramos-Escobar N. (2019). Neuroinflammation as a common feature of neurodegenerative disorders. *Front. Pharmacol.* 10:1008. 10.3389/fphar.2019.01008 31572186 PMC6751310

[B12] Hanahan D. Weinberg R. A. (2011). Hallmarks of cancer: the next generation. *Cell* 144 646–674. 10.1016/j.cell.2011.02.013 21376230

[B13] Holohan K. N. Lahiri D. K. Schneider B. P. Foroud T. Saykin A. J. (2012). Functional microRNAs in Alzheimer’s disease and cancer: differential regulation of common mechanisms and pathways. *Front. Genet.* 3:323. 10.3389/fgene.2012.00323 23335942 PMC3547332

[B14] Itzhaki R. F. Lathe R. Balin B. J. Ball M. J. Bearer E. L. Braak H. (2016). Microbes and Alzheimer’s disease. *J. Alzheimers. Dis.* 51 979–984. 10.3233/JAD-160152 26967229 PMC5457904

[B15] Kitdumrongthum S. Metheetrairut C. Charoensawan V. Ounjai P. Janpipatkul K. Panvongsa W. (2018). Dysregulated microRNA expression profiles in cholangiocarcinoma cell-derived exosomes. *Life Sci.* 210 65–75. 10.1016/j.lfs.2018.08.058 30165035

[B16] Kleaveland B. Shi C. Y. Stefano J. Bartel D. P. (2018). A network of noncoding regulatory RNAs acts in the mammalian Brain. *Cell* 174 350.e317–362.e317. 10.1016/j.cell.2018.05.022 29887379 PMC6559361

[B17] Kumar P. Dezso Z. MacKenzie C. Oestreicher J. Agoulnik S. Byrne M. (2013). Circulating miRNA biomarkers for Alzheimer’s disease. *PLoS One* 8:e69807. 10.1371/journal.pone.0069807 23922807 PMC3726785

[B18] Lane C. A. Hardy J. Schott J. M. (2018). Alzheimer’s disease. *Eur. J. Neurol.* 25 59–70. 10.1111/ene.13439 28872215

[B19] Lasek W. Zagozdzon R. Jakobisiak M. (2014). Interleukin 12: still a promising candidate for tumor immunotherapy? *Cancer Immunol. Immunother.* 63 419–435. 10.1007/s00262-014-1523-1 24514955 PMC3994286

[B20] Li J. Lin H. Sun Z. Kong G. Yan X. Wang Y. (2018). High-throughput data of circular RNA profiles in human temporal cortex tissue reveals novel insights into temporal lobe epilepsy. *Cell Physiol. Biochem.* 45 677–691. 10.1159/000487161 29428937

[B21] Li J. H. Liu S. Zhou H. Qu L. H. Yang J. H. (2014). starBase v2.0: decoding miRNA-ceRNA, miRNA-ncRNA and protein-RNA interaction networks from large-scale CLIP-Seq data. *Nucleic Acids Res.* 42 D92–D97. 10.1093/nar/gkt1248 24297251 PMC3964941

[B22] Lin R. J. Xiao D. W. Liao L. D. Chen T. Xie Z. F. Huang W. Z. (2012). MiR-142-3p as a potential prognostic biomarker for esophageal squamous cell carcinoma. *J. Surg. Oncol.* 105 175–182. 10.1002/jso.22066 21882196

[B23] Love M. I. Huber W. Anders S. (2014). Moderated estimation of fold change and dispersion for RNA-seq data with DESeq2. *Genome Biol.* 15:550. 10.1186/s13059-014-0550-8 25516281 PMC4302049

[B24] Mills J. D. van Vliet E. A. Chen B. J. Janitz M. Anink J. J. Baayen J. C. (2020). Coding and non-coding transcriptome of mesial temporal lobe epilepsy: critical role of small non-coding RNAs. *Neurobiol. Dis.* 134:104612. 10.1016/j.nbd.2019.104612 31533065

[B25] Moremen K. W. Robbins P. W. (1991). Isolation, characterization, and expression of cDNAs encoding murine alpha-mannosidase II, a Golgi enzyme that controls conversion of high mannose to complex N-glycans. *J. Cell Biol.* 115 1521–1534. 10.1083/jcb.115.6.1521 1757461 PMC2289207

[B26] Morris J. C. (1993). The clinical dementia rating (CDR): current version and scoring rules. *Neurology* 43 2412–2414. 10.1212/wnl.43.11.2412-a 8232972

[B27] Musicco M. Adorni F. Di Santo S. Prinelli F. Pettenati C. Caltagirone C. (2013). Inverse occurrence of cancer and Alzheimer disease: a population-based incidence study. *Neurology* 81 322–328. 10.1212/WNL.0b013e31829c5ec1 23843468

[B28] Nikolac Perkovic M. Pivac N. (2019). Genetic markers of Alzheimer’s disease. *Adv. Exp. Med. Biol.* 1192 27–52. 10.1007/978-981-32-9721-0_3 31705489

[B29] Nudelman K. N. H. McDonald B. C. Lahiri D. K. Saykin A. J. (2019). Biological hallmarks of cancer in Alzheimer’s disease. *Mol. Neurobiol.* 56 7173–7187. 10.1007/s12035-019-1591-5 30993533 PMC6728183

[B30] Park S. K. Park Y. S. Ahn J. Y. Do E. J. Kim D. Kim J. E. (2016). MiR 21-5p as a predictor of recurrence in young gastric cancer patients. *J. Gastroenterol. Hepatol.* 31 1429–1435. 10.1111/jgh.13300 26824898

[B31] Piwecka M. Glazar P. Hernandez-Miranda L. R. Memczak S. Wolf S. A. Rybak-Wolf A. (2017). Loss of a mammalian circular RNA locus causes miRNA deregulation and affects brain function. *Science* 357:eaam8526. 10.1126/science.aam8526 28798046

[B32] Pu C. Huang H. Wang Z. Zou W. Lv Y. Zhou Z. (2018). Extracellular vesicle-associated mir-21 and mir-144 are markedly elevated in serum of patients with hepatocellular carcinoma. *Front. Physiol.* 9:930. 10.3389/fphys.2018.00930 30065664 PMC6056643

[B33] Savage J. E. Jansen P. R. Stringer S. Watanabe K. Bryois J. de Leeuw C. A. (2018). Genome-wide association meta-analysis in 269,867 individuals identifies new genetic and functional links to intelligence. *Nat. Genet.* 50 912–919. 10.1038/s41588-018-0152-6 29942086 PMC6411041

[B34] Shafi O. (2016). Inverse relationship between Alzheimer’s disease and cancer, and other factors contributing to Alzheimer’s disease: a systematic review. *BMC Neurol.* 16:236. 10.1186/s12883-016-0765-2 27875990 PMC5120447

[B35] Shannon P. Markiel A. Ozier O. Baliga N. S. Wang J. T. Ramage D. (2003). Cytoscape: a software environment for integrated models of biomolecular interaction networks. *Genome Res.* 13 2498–2504. 10.1101/gr.1239303 14597658 PMC403769

[B36] Shi S. Gu S. Han T. Zhang W. Huang L. Li Z. (2020). Inhibition of MAN2A1 enhances the immune response to Anti-PD-L1 in human tumors. *Clin. Cancer Res.* 26 5990–6002. 10.1158/1078-0432.CCR-20-0778 32723834 PMC8500537

[B37] Snyder H. M. Ahles T. Calderwood S. Carrillo M. C. Chen H. Chang C. H. (2017). Exploring the nexus of Alzheimer’s disease and related dementias with cancer and cancer therapies: a convening of the Alzheimer’s association & Alzheimer’s drug discovery foundation. *Alzheimers Dement* 13 267–273. 10.1016/j.jalz.2016.11.002 27998721 PMC5548424

[B38] Sochocka M. Donskow-Lysoniewska K. Diniz B. S. Kurpas D. Brzozowska E. Leszek J. (2019). The gut microbiome alterations and inflammation-driven pathogenesis of Alzheimer’s disease-a critical review. *Mol. Neurobiol.* 56 1841–1851. 10.1007/s12035-018-1188-4 29936690 PMC6394610

[B39] Tugues S. Burkhard S. H. Ohs I. Vrohlings M. Nussbaum K. Vom Berg J. (2015). New insights into IL-12-mediated tumor suppression. *Cell Death. Differ.* 22 237–246. 10.1038/cdd.2014.134 25190142 PMC4291488

[B40] Vo J. N. Cieslik M. Zhang Y. Shukla S. Xiao L. Zhang Y. (2019). The landscape of circular RNA in cancer. *Cell* 176 869.e813–881.e813. 10.1016/j.cell.2018.12.021 30735636 PMC6601354

[B41] Vom Berg J. Prokop S. Miller K. R. Obst J. Kalin R. E. Lopategui-Cabezas I. (2012). Inhibition of IL-12/IL-23 signaling reduces Alzheimer’s disease-like pathology and cognitive decline. *Nat. Med.* 18 1812–1819. 10.1038/nm.2965 23178247

[B42] Wang M. Beckmann N. D. Roussos P. Wang E. Zhou X. Wang Q. (2018). The Mount Sinai cohort of large-scale genomic, transcriptomic and proteomic data in Alzheimer’s disease. *Sci. Data* 5:180185. 10.1038/sdata.2018.185 30204156 PMC6132187

[B43] Xia X. Jiang Q. McDermott J. Han J. J. (2018). Aging and Alzheimer’s disease: comparison and associations from molecular to system level. *Aging Cell* 17:e12802. 10.1111/acel.12802 29963744 PMC6156542

[B44] Xu J. Z. Shao C. C. Wang X. J. Zhao X. Chen J. Q. Ouyang Y. X. (2019). circTADA2As suppress breast cancer progression and metastasis via targeting miR-203a-3p/SOCS3 axis. *Cell Death Dis.* 10:175. 10.1038/s41419-019-1382-y 30787278 PMC6382814

[B45] Yan D. Dong W. He Q. Yang M. Huang L. Kong J. (2019). Circular RNA circPICALM sponges miR-1265 to inhibit bladder cancer metastasis and influence FAK phosphorylation. *EBioMedicine* 48 316–331. 10.1016/j.ebiom.2019.08.074 31648990 PMC6838432

[B46] Yan J. Smyth M. J. Teng M. W. L. (2018). Interleukin (IL)-12 and IL-23 and their conflicting roles in cancer. *Cold Spring Harb. Perspect. Biol.* 10:a028530. 10.1101/cshperspect.a028530 28716888 PMC6028064

[B47] Zhang Q. Guo S. Zhang X. Tang S. Shao W. Han X. (2015). Inverse relationship between cancer and Alzheimer’s disease: a systemic review meta-analysis. *Neurol. Sci.* 36 1987–1994. 10.1007/s10072-015-2282-2 26248482

[B48] Zhao X. Cai Y. Xu J. (2019). Circular RNAs: biogenesis, mechanism, and function in human cancers. *Int. J. Mol. Sci.* 20:3926. 10.3390/ijms20163926 31412535 PMC6720291

